# Supplementary figures and images for: A miRNome analysis at the early postmortem interval
Source: PeerJ. 2023 Jun 7;11:e15409. doi: 10.7717/peerj.15409 (PMC10257396; doi:10.7717/peerj.15409)

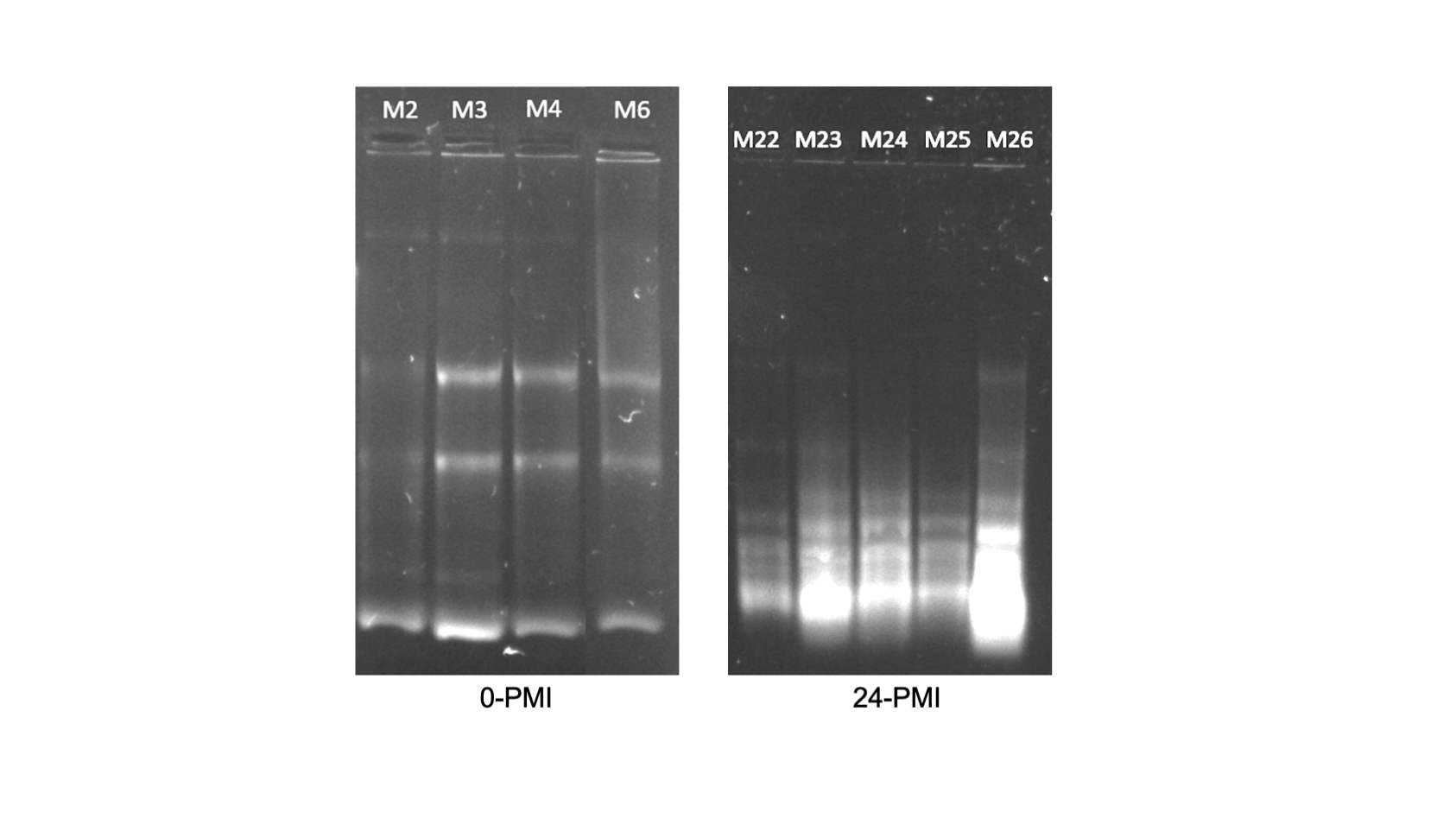

Supplement: Supplemental Information 4 [file peerj-11-15409-s004.jpg]

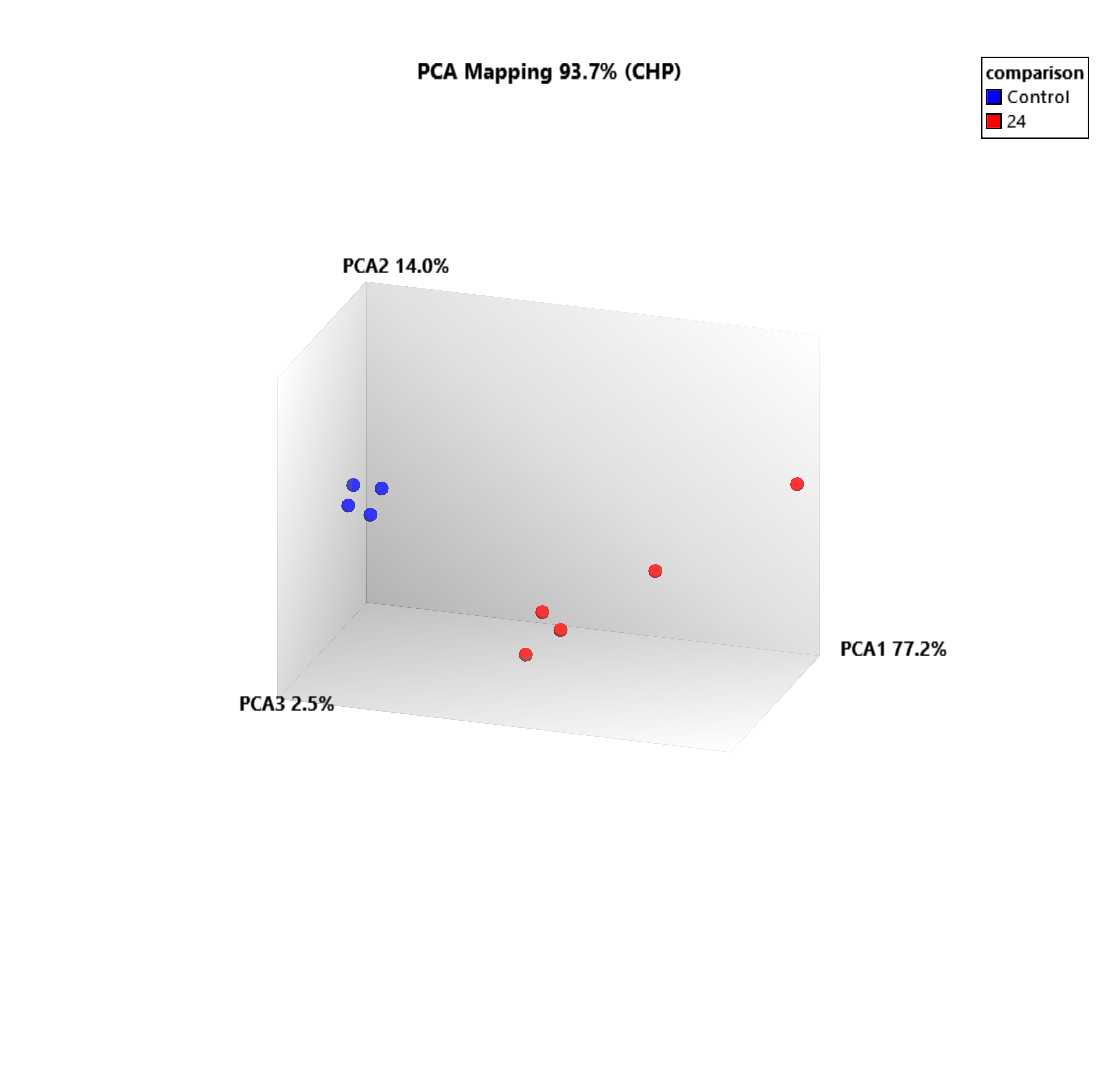

Supplement: Supplemental Information 5 [file peerj-11-15409-s005.png]
