# Supplementary material for: A miRNome analysis at the early postmortem interval
Source: PeerJ. 2023 Jun 7;11:e15409. doi: 10.7717/peerj.15409 (PMC10257396; doi:10.7717/peerj.15409)
Supplement: Supplemental Information 9 — The sequence of the oligos used to analyze the expression of TGFBR2, SIRT1, BMF and GAPDH using RT-PCR [file peerj-11-15409-s009.docx]

| mRNA | Oligo Forward 5´ | Oligo Reverse 5´ |
| --- | --- | --- |
| BMF | AGC CCG CTG GAG TTT GTC | CTC CGA GTC GTG ATG CCA G |
| Sirt1 | TGT TTC CTG TGG GAT ACC TGA | TGA AGA ATG GTC TTG GGT CTT T |
| Tgfbr2 | CGT GTG GAG GAA GAA CGA CA | CGT GGG AGA AGT GGC ATC TT |
| GAPDH | GGG TGT GAA CCA CGA GAA ATA | AGT TGT CAT GGA TGA CCT TGG |
