# Supplementary material for: A miRNome analysis at the early postmortem interval
Source: PeerJ. 2023 Jun 7;11:e15409. doi: 10.7717/peerj.15409 (PMC10257396; doi:10.7717/peerj.15409)
Supplement: Supplemental Information 10 [file peerj-11-15409-s010.doc]

**Miame Checklist**

# **1. Experiment Design**

Type of Experiment: control group vs 24h of postmortem interval group

Experimental variables: Two groups of rats: control group (without contusion, n=4)

24h of postmortem interval group (n=5)

Hybridization array: Affymetrix GeneChip miRNA 4.0 Array (Affymetrix, Santa Clara, CA, USA)

Tissue used for slide: Skeletal muscle

other variables: In total 9 adult male Wistar rats, all with an average weight of 200 +/- 20 gr

# **2. Array design**

Array series: Amplified and biotinylated sense-strand DNA targets of miRNAs transcripts.

Deconvoluted spot list with gene names: Deconvoluted spot list is available at <https://www.thermofisher.com/mx/es/home/life-science/microarray-analysis.html>

Array type: All Organisms, Human, Mouse, Rat (30 424 probes)

Slide type (and coating): The array consists of a square glass that contains an array of oligonucleotides that will be hybridized with the labeled cDNA.

# **3. Samples**

Source of the sample: 24h of postmortem interval group, femoral skeletal muscle sample was taken after 24h of post-mortem interval (n=5).

For control group, femoral skeletal muscle sample were taken immediately after euthanasia.

Postmortem interval rat model:

A total of 9 adult male Wistar rats was selected for the study; all with an average weight of 200 +/−20 gr. These rats were sorted into two groups, which correspond to the 0 and 24 h of post-mortem interval (h-PMI). The 0 h-PMI was considered the control group. The rats from 24 h-PMI group, were euthanized by cervical dislocation and placed in a Binder KBW 240™ climatic chamber with a constant temperature of 25 °C. After the PMI time elapsed in this group, the presence of internal and external morphological changes was evaluated on every rat. Once the evaluation was performed, 200 mg of femoral muscle sample was obtained and stored at −80 °C until analysis. The rats from the control group were euthanized by cervical dislocation, and muscle samples were taken immediately and stored at −80 °C until analysis. As in the other PMI-groups, control group rats were externally and physically evaluated for the presence of cadaveric signs.

# **4. Hybridizations**

Hybridization protocol: Hybridizations were performed for 17 hours at at 45°C and 60 rpm using GeneChip Hybridization Oven 645.

Washing Protocol: A standard series of high stringency washes were performed using GeneChip Fluidics Station 450.

# **5. Measurement**

Scanning and software: GeneChip Scanner 3000 7G (Packard Bioscience) was used and it was controlled by the software named GeneChip Command Console Software.

Data Files: 9 raw data files in CEL file

Type of Data: Raw data in in CEL file format

Data Transformation: Raw data of CEL files were normalized and transformed in to CHP files by Robust Multi-chip Analysis (RMA) setting the value of Detected Above Background (DABG) to 0.05 using Affymetrix Transcriptome Analysis Console software.

# **6. Normalization controls**

For comparison, control group and 24h of postmortem interval groups were compared and the changes of gene expression were shown as mean Fold Change (FC), considering miRNAs over or down ex-pressed with thresholds above >2 or below <2, respectively. Significant dysregulated miRNAs were those with p-value less than 0.01 and a False Discovery Rate value less than 0.05.
